# The Differential Diagnosis of Swelling in the Breast

**Published:** 1912-06

**Authors:** Charles A. Morton

**Affiliations:** Professor of Surgery in the University of Bristol; Senior Surgeon to the General Hospital and the Children's Hospital


### THE DIFFERENTIAL DIAGNOSIS OF SWELLING IN THE BREAST.

BY

CHARLES A. MORTON, F.R.C.S.,
*Professor of Surgery in the University of Bristol;*
*Senior Surgeon to the General Hospital and the Children's Hospital.*

---

THERE are certain chronic swellings in the breast which we frequently meet with—cysts, fibro-adenoma, localised sclerosing mastitis, and scirrhus. A breast swelling may, of course, be a chronic abscess, or a sarcoma, or a duct carcinoma, or the soft form of glandular carcinoma, or some other very rare form of tumour; but it will probably be one of the first four forms I have mentioned. Acute suppuration in the breast is hardly likely to lead to any serious difficulty in diagnosis, but we must recognise the fact that now and again, with a rapidly-growing malignant tumour of the breast there may be quite marked redness of the overlying skin and œdema, so that on first looking at the breast we may feel sure the patient has an abscess in it. I remember some years ago a woman who came up as an out-patient at the Hospital, with what looked like an abscess of the breast. The skin was bright red and œdematous, but it overlaid a firm malignant growth. I also saw in consultation with one of my colleagues a few years ago a woman aged 39, with a red brawny condition of the skin overlying the breast. It certainly looked inflammatory, and before she came to the Hospital her own doctor had incised it, thinking it was an abscess. Indeed, when I examined her there was a soft fluctuating area which strongly suggested abscess. But there was no pus, and the whole condition was one of malignant growth. About the same time I also saw another case of a woman aged 49, who had a fluctuating boss protruding from a large swelling of the breast, and over it the skin was red. However, only serous fluid had been withdrawn by an exploring needle. It was found when removed to be a carcinoma, with a degeneration cyst. Quite recently I have had another very striking case of this kind. A woman, aged 65, was sent to me with a tumour of the breast of several months' duration, and its superficial part was soft and fluctuating. While waiting for admission she came to see me again, with the skin overlying it bright red and slightly œdematous, giving it quite the appearance of a pointing abscess. Two medical men and five senior students who examined her were quite sure she had an abscess in the breast. But she had enlarged and hard glands in the axilla, marked retraction of the nipple, and serous oozing from the nipple. I removed one of the axillary glands to see if it contained growth, but did not find any. I then (at the same operation) removed the breast, and after removal cut it open, and found a soft white malignant tumour, with a small degeneration cyst, containing clear serous fluid. I then completed the usual operation for cancer of the breast. I might have simply dissected out the tumour, and then cut into it, but it was so large that this would have been a removal of half the breast, and I should probably have obstructed the main ducts of the other half by the contraction of the scar tissue, and therefore I removed the whole breast, which at her age was no great loss to her. I am sure there is danger in putting an exploring syringe needle into a tumour you suppose may be malignant, as cancer cells may be diffused through the puncture. The danger, perhaps, is not great if you are prepared at once to remove the tumour if necessary. but there must even then be some slight risk of dissemination. To cut into the swelling before removal involves greater risk of dissemination. The safe way is to cut out the suspicious lump, and then cut it across. Even if it is not malignant, its excision is generally the best treatment. In this case if it had turned out to be a chronic abscess, excision would have been the best treatment. When, however, the swelling lies under the nipple, so that its excision would damage the main ducts, or when it involves a very large portion of the breast, the removal of the breast is indicated, and the pectoral muscle and axillary contents, and more skin, can be removed if the tumour is found to be malignant. The tumour in this case was clearly malignant, though it was certainly not scirrhus, and the probability seems to be that it was an endothelioma, but it would probably be called a sarcoma by some pathologists. No growth was discovered in the enlarged axillary glands.

Before I further consider the differential diagnosis of swelling actually in or on the breast, I must draw attention to the fact that disease of a neighbouring structure may simulate a tumour in the breast. Those of us who used to read Erichsen's *Surgery* will remember that he relates how, in University College Hospital, a man was supposed to be suffering from scirrhus of the breast. The lump in the breast was explored, and was still considered to be scirrhus. The breast was therefore removed, but on further examination after removal a small cavity was discovered in the pectoral muscle, and at the bottom of this a small sequestrum was found in the rib. It was, in fact, a case of "quiet" necrosis of a rib, with fibroid thickening in the overlying breast. The breast may be greatly pushed forward by an abscess beneath, and it is important not to mistake this for enlargement of the breast itself. Such an abscess, if chronic, may of course be due to tuberculous disease of a rib. An acute abscess may, however, be sub-mammary, and then, together with the pushing forward of the breast, there would probably be œdema at its base, but it might be quite difficult to get evidence of fluctuation. Such an abscess might arise from suppuration starting in a part of the breast just overlying the pectoral muscle. I once had a case of sub-mammary abscess, with much surrounding induration, sent to me as a case of scirrhus deep in the breast. An indistinct feeling of fluctuation, with one hand on the breast and the other on the edge of the sub-pectoral swelling at one particular spot, suggested the presence of pus. And a very remarkable case came under my care in the Hospital some years ago, in which a cyst in the pectoral muscle simulated a tumour of the breast, and I proceeded to remove it, thinking I was dealing with a tumour of the breast, but I cut through the breast and found no tumour. I then cut through the pectoral fascia beneath and found an hydatid cyst lying in the substance of the pectoral muscle.

It is, I suppose, well known that a simple serous retention cyst of the breast may be so hard from tension as to feel like a hard solid growth. Numerous instances are on record in which this mistake has occurred. The tense cyst does not even feel elastic, much less fluctuating. Hence it is never wise to give a definite opinion that a hard lump in the breast is necessarily scirrhus. I have removed such lumps, which had been confidently diagnosed as scirrhus, and found them tense cysts, and I feel very strongly that it is never wise to perform the complete operation for cancer of the breast without first excising such a lump, in case it turns out to be an innocent tumour. I used to cut into the tumour, but now I always excise it first. The only possible risk in doing so is diffusion of cancer cells lying in the lymphatic vessels between it and the glands by their division; but if the growth is found to be cancer all the surrounding tissue will then be excised, and if the cancer cells have already invaded the lymphatic vessels we must regard the growth as already diffused beyond what is visible and palpable, and it can matter little if we cut through such infected lymphatics. It is certainly better to cut out the growth, and then cut into it, than to subject the patient to so extensive an operation as that for mammary cancer without any need.

But it is not only scirrhus that a tense cyst may simulate. I have known a tense cyst projecting from the breast mistaken for fibro-adenoma by several surgeons who examined the case on two occasions. In one the supposed fibro-adenoma was found on removal to be a cyst, and in the other the tumour, which was the size of a hen's egg, almost completely disappeared.

Not only may a cyst when tense feel like a solid growth, but a chronic abscess may also. Moreover, a chronic abscess may simulate a scirrhous growth because it may be associated with some retraction of the nipple. Overlying œdema may, however, suggest chronic abscess. In a clinical lecture on the differential diagnosis of swelling of the breast which I published in the *Clinical Journal* in 1897, IX., 327, I referred to two cases of chronic abscess of the breast under my care which simulated solid tumours. One was the case of a young woman 23 years of age who came to the Hospital with a firm, round, finely-lobular swelling, the size of a small hen's egg, deeply situated in the breast. There was no fluctuation—not even an elastic feeling on firm pressure. It had been noticed for a month, and there had been shooting pains in it. Both nipples looked retracted, but were really undeveloped. She was single, and had never suckled. An incision evacuated several drachms of watery pus, which had been contained in a smooth walled cavity. There was not much induration of the breast tissues around, and the solid feeling was due to the great tension within the cavity. The other case was that of a woman aged 43, who came to the Hospital with a small lump in the breast which she had noticed three weeks before, and it had been quite painless. There was a hard, smooth, round nodule, the size of a small marble, projecting from the breast close to the nipple. It was not tender, and there was no redness of the skin over it. As in the other case, there was no fluctuation, and it was not even elastic or firm on pressure. The skin was a little drawn in over it. It was found to contain thick pus. Some surgical writers state that chronic abscess usually occurs in connection with lactation, but statistics do not bear out this statement. In neither of these two cases was the patient suckling.

But not only is a fluid swelling not always fluctuating, but a fluctuating swelling is not always a cyst or abscess. A solid growth (usually malignant) may be soft enough to fluctuate. I hope it is now well recognised that a soft solid may fluctuate just in the same way that a fluid may. Fluctuation is not a "fluid wave" as it is sometimes said to be. It is nothing more than the alteration in the shape of a solid, or a collection of air, or fluid, made by pressure upon it. You press down the substance at one point, and with the finger of the other hand you feel it rise up at another. This is all fluctuation is, and you can never get it more perfectly than in a soft lipoma. The mistake one is apt to make is that one sometimes moves the body as a whole, and then imagines that one has altered its shape by pressure. For instance, you press a solid gland with one finger so that it moves as a whole, and you will get the pressure conveyed to the other finger, and may think you have fluctuation.

We must never forget that we may have a cyst of the breast combined with a solid malignant growth. Such a combination may occur in sarcoma, and then the cyst is usually part of the tumour formation. But in carcinoma, occasionally, you may get a degeneration cyst. The tumour tissue breaks down, and a serous cyst is found, without any distinct capsule, but with a wall of tumour tissue. And such a malignant growth may present mainly as a cyst. I have in more than one instance made the diagnosis by the exploring syringe. After drawing off the serous fluid, I have found that some portion of the swelling remained, and on operating have found the combination of carcinoma with a degenerative cyst. But I have come to the conclusion that if there is reason to suspect the cyst may be only part of a malignant growth, it is better to remove the tumour, and then cut it across and thus determine its nature, for I think malignant cells may be disseminated through the needle track, together with fluid from the cyst.

A soft, elastic, non-fluctuating swelling projecting even from the surface of the breast is quite likely to be a common fibro-adenoma. But a fibro-adenoma may also be quite firm. Such a growth, *i.e.* a firm growth projecting well from the surface of the breast, without any tethering of the overlying skin, or retraction of the nipple, or any enlarged glands, is almost certain to be diagnosed as a fibro-adenoma; but it may turn out to be carcinoma. I had one very striking case of this kind. A woman only 32 came under my care with a nodule the size of a large marble projecting from the surface of a thin breast. It was firm but not really hard. There was no dimpling or tethering of the overlying skin. As it was at the periphery of the breast, the absence of retraction of the nipple was of no importance. There were no glands. I felt sure it was a fibro-adenoma, and even felt doubtful if it should be removed; but I did remove it, and it turned out to be a scirrhus nodule, typical both to the naked eye and on microscopic section.

It is often stated in the books that a fibro-adenoma "moves on" or "in" the breast, and that this is a valuable diagnostic sign. I have removed a good many fibro-adenomata of the breast, but I have never yet seen one that could be moved on the breast: they are always sessile on its surface. In order that it may move on the breast the tumour must obviously be either pedunculated or lying loose on it. Neither condition exists. The idea that a fibro-adenoma moves on the breast is due to the ease with which when you grasp the tumour you move the underlying breast tissue with it, without recognising that you do so. The tumour forms such an excellent handle you can move the surrounding portion of the breast freely if you grasp it; and there is nothing that will give you evidence of movement of the surrounding breast tissue, and hence you do not realise that you are moving it also, but imagine that you are moving the tumour on it, or in it. I think it is because a fibro-adenoma is a surface tumour that one is apt to get the impression that it moves on the breast. When a tumour is imbedded in the breast, as a cyst often is, it is easier to recognise that one is moving the surrounding breast tissue with it.

An ill-defined outline of a swelling of the breast certainly suggests that it may be sclerosing mastitis, rather than a tumour. It is sometimes stated that you cannot feel such a swelling with the flat of the fingers, but only on taking up the breast between the thumb and finger. A swelling of such a character could certainly hardly be anything but sclerosing mastitis; but we must remember that every now and again a lump of sclerosing mastitis may be so perfectly well defined and so hard, that without exploration it may not be possible to say it is not scirrhus. This is another reason why we should always cut out a hard lump in the breast which we think is cancer, and then cut it across before proceeding further with the operation. We must remember that cysts and sclerosis mastitis often exist together in the same breast. The multiplicity of hard lumps would certainly suggest that they might be sclerosing mastitis rather than malignant growths, but I have seen several cases where two scirrhous growths were present in the same breast. In a case in which we were in doubt as to the diagnosis between sclerosing mastitis and scirrhus, after cutting across the excised lump, an immediate microscopic section might settle the question; but the microscopic diagnosis between sclerosing mastitis and cancer, in an early stage, is notoriously different, and it might be that only after a very careful examination of a large number of sections taken from different parts of the lump that a definite diagnosis could be made, and even then it might not be quite certain. Of course, if we had any doubt after examining an immediate microscopic section we should act wisely in performing the complete operation for cancer, recognising the fact that cancer occurs in quite young women—under 30—sometimes. We must never allow age to settle the diagnosis in a doubtful case.

Scirrhous cancer usually occurs as a hard lump—often stony hard—and it is frequently evident that it is in the breast, but sometimes it may seem only to project from it, and in that way to resemble a fibro-adenoma. A very hard solid tumour is nearly always scirrhus, but as I have already pointed out a tense cyst may feel quite hard. I once, however, operated for removal of a very hard growth which I expected to find was scirrhus, but found an ossifying sarcoma. I have fully recorded the case in the *Transactions* of the Pathological Society.

A scirrhous growth is sometimes irregular and even nodular, and when the overlying skin is moved, it is sometimes found to be tacked down, as it were, to the lump, or the nipple may be retracted. It is necessary to be cautious in drawing conclusions from an apparently retracted nipple. We must always make sure it has ever been protruded, for sometimes what you think is a retracted nipple is really a long-standing abnormality. If no glands can be felt in the axilla in a case of suspected cancer of the breast, it is no evidence that the growth is not cancer. I frequently have operated on cancer of the breast without palpably enlarged axillary glands. On the other hand, distinctly hard enlarged axillary glands, with a doubtful lump in the breast, are of considerable diagnostic importance. It is well to remember that it is the pectoral group—the group lying just under the border of the great pectoral muscle—which is likely to be first affected, though lymphatics may pass from the upper segment of the breast direct to the highest axillary glands—the sub-clavicular group lying just above the upper border of the pectoralis minor. Whether a growth of the breast seems to protrude from its surface will very much depend on the amount of fat with which the breast is incorporated.

A growth which really projects well from its surface may yet feel imbedded in it, for it may be buried in the surrounding fat, which it is impossible to recognise apart from the breast. As I have already pointed out in speaking of cysts, if we are in doubt as to the nature of any swelling of the breasts which might possibly be cancer, it is best to excise it, and then cut it across, and if necessary have an immediate microscopic section made, being prepared to do the complete operation for cancer if it should be shown to be malignant. If it is a question whether it may not be a tense cyst, it might be wise to see the patient again after a short interval, to try again for any evidence of fluctuation; but it is not wise to leave indefinitely doubtful swellings in the breast which may be cancer, in the hope that in time the nature of the swelling may be evident. The patient has probably already delayed showing it to her doctor, because it gave her no pain, and therefore she thinks it cannot be anything serious, whereas one seldom finds any pain with early scirrhus.

Sometimes we are consulted by middle-aged women about a puckering in the skin over some small area of a fat breast, and we can feel no lump beneath. But such a condition is most suspicious of a small scirrhous growth deep in the breast, buried under the fat; and in such cases the breast should always be explored with a view to the performance of the complete operation for cancer. I have seen more than one case of this kind, and found a small scirrhous growth imbedded in a fat breast on exploration.

What is the diagnostic value of serous discharge from the nipple without any swelling as a sign of cancer? I do not think even in a middle-aged person it is suspicious enough to make it wise to remove the breast, but in addition to a swelling of the breast it is, I think, an unfavourable sign. Discharge of blood from the nipple, even without any tumour, I should regard as more suspicious than serous oozing, but an innocent papilloma growing in a cyst near the nipple, too small and too deeply placed for detection, might account for it. Bleeding from the nipple occurs in duct carcinoma, but there would certainly be a palpable tumour, and it is quite one of the uncommon forms of breast tumour. I should certainly watch very carefully the breast in a case of bleeding from the nipple for the appearance of any swelling, but I do not think I should at once excise it unless there was also a lump in it.

There is so much difference of opinion as to the nature of Paget's disease of the nipple that it is difficult to write anything definite about it. What Sir J. Paget originally described was an intractable eczema of the nipple, which was very often found to be associated with ordinary glandular cancer in the breast. And yet the nipple is sometimes destroyed by ulceration, which shows that the morbid process is certainly more than an eczema. The term "malignant dermatitis" is a bad one. It must be either one or the other, either dermatitis or the ulceration of a malignant growth, though possibly the latter might be engrafted on the former. I have had one case under my care in which there was a raw, scabbed condition of the nipple, so that when the scab was removed, the nipple looked like a raspberry, and there was a hard lump unconnected with the nipple in the breast, and hard axillary glands. The woman was 69 years of age. It was a primary epithelioma of the nipple, and the lump in the breast was secondary epithelioma, and there was secondary growth in the glands. The true nature of the case was only discovered by microscopic examination of the parts removed at the operation. When I cut into the lump in the breast at the time of the operation I was much puzzled by its appearance. I had never seen such a tumour in the breast. It was a firm white growth, and contained numerous very small, smooth lined, cavities, which contained material like dirty cream cheese. Such a substance is sometimes found in secondary epitheliomatous glands, but not usually in little cavities with such definite walls. It is, of course, due to a fatty degeneration of the growth.

If a rapidly growing tumour of the breast has not the character of scirrhus, if, in fact, it is soft or elastic or perhaps bossed, can we diagnose its nature before operation—as to whether it is encephaloid carcinoma, a duct cancer, sarcoma or endothelioma ? In sarcoma it is stated that the glands are not usually affected. But in the *Transactions* of the Pathological Society some years ago I recorded a case of sarcoma of the breast, with marked secondary growth in the axillary glands, and on the other hand I should certainly clean out the axillary contents in operation on such a tumour, whether I felt enlarged glands or not, for I think it is possible that with even a rapidly growing encephaloid cancer there might possibly be no glands palpable, though this would of course be unlikely. In duct cancer there is often a bloody discharge from the nipple. I really do not think we could determine the exact nature of a malignant tumour of the character I have just described. It is certain that fluctuating bosses on it may be soft solid growth, and not cysts, as we are likely to suppose them to be. In dealing with such a tumour, if it was too large to cut out and examine at the time of operation, I am sure the best plan would be to do the complete operation for cancer. If the growth is shown by careful examination after removal to be a duct carcinoma, we may feel very hopeful as to prognosis. Indeed, it is a question whether it is necessary to remove the glands and the pectoral muscle in such cases, but perhaps it would be wise to do so, even if we were able to make the diagnosis of duct cancer with certainty before operation.

It is impossible to write about the diagnosis of swellings in the breast without reference to the possible change from an innocent into a malignant tumour. There is no doubt that often swellings which have been regarded as innocent tumours have been found to be malignant when removed, and again and again it has been stated that they have changed from one into the other. This is, of course, quite possible. The history we get sometimes, that for a great many years a swelling has existed in the breast of much the same size, and ultimately taken on rapid growth, and is found on removal to be malignant, certainly suggests such a transference. When such a tumour is removed there may be no sign of anything but the malignant form of growth, all sign of an innocent form, if it ever existed, may have disappeared. If one did get a growth with such a history, and it showed innocent growth in one part and malignant in another, it would of course be conclusive evidence. But because a lump in the breast has been diagnosed as a patch of sclerosing mastitis or a fibro-adenoma, and watched as such for many months, or even a year or two, and is then found on removal to be malignant, is not to my mind sufficient proof that it was ever an innocent growth. We know how variable is the rate of growth of malignant tumours, and how easy it is to make a mistake in diagnosis. If we cannot keep what we diagnose as an innocent tumour of the breast under observation, so as to examine it every few months, it seems safer to remove it, for knowing that a lump already existed, any change in its character would not be recognised by the patient herself, and possibly any suspicious sign which might develop might not be recognised by some other medical man the patient might happen to consult.

With regard to the multiplicity of breast swellings, I think we may say that cysts are very often multiple, and in both breasts; patches of sclerosis mastitis frequently are multiple, and that it is not very rare to meet with more than one fibro-adenoma in the breast. As I have already mentioned, I have seen several cases of two scirrhous growths in the same breast, so that we must not on account of there being more than one primary growth entirely negative carcinoma. I have several times operated on cases in which there was a cancerous growth in both breasts at the same time.

This paper deals with swellings in the breast, and not with general enlargement of the breast, which may be due to simple hypertrophy, and then both breasts may be greatly enlarged, and in some cases they are very painful. But it is important to point out that the whole breast may seem enlarged, when really it is a large fibro-adenoma growing in it. I removed such a tumour some years ago from a case I at first thought was one of general enlargement of the breast.

We may now perhaps with advantage consider the value of separate signs. Retraction of the nipple (not undeveloped nipple), or retraction or tethering of the overlying skin, is an important sign of scirrhus, but has been known to occur in chronic abscess, or even simply with sclerosing mastitis. As I have already pointed out, puckering of the overlying skin may be the only sign of a deeply-buried scirrhous growth. In cancer we may have quite hard enlarged glands, but the glands may also be decidedly enlarged in chronic abscess, and they are said to be particularly so in tuberculous disease. But we must clearly recognise the fact that we may not find any enlarged glands in early cancer. Redness and œdema of the overlying skin is certainly suggestive of suppuration, but both conditions may be present with a non-suppurating malignant growth. The hardness of a lump is suggestive of scirrhus, but a very tense cyst may be as hard. What is sometimes spoken of as the weight of a tumour of the breast is supposed to have some diagnostic importance, but we must remember that a fat breast itself is quite heavy. Mobility of any tumour in the breast, or on it, I do not believe exists, except in very rare cases in which it happens to be pedunculated. I have never seen such a tumour of the breast. With regard to the one symptom, pain, it may be said that it is not usually present in early cancer, and is much more often present in a tense cyst. It is most important to remember this, and not to be misled, as the patient probably is, by thinking that because there is no pain it is not a serious condition.

I have not myself had a case under my care of tuberculous disease of the breast, and I do not see how it would be possible to make a diagnosis of a solid tuberculous deposit. Enlargement of the glands, which is said to be marked in tuberculous disease might suggest cancer. If it did, excision of the lump would be excellent treatment, and the diagnosis could then be made. Any chronic abscess might of course be tuberculous.

There is, I think, nothing special to be said about the diagnosis of swellings in the male breast. We must remember that scirrhus occurs in the male as well as the female breast. I had some years ago a very remarkable case of syphilitic sclerosing mastitis of the male breast. The patient had typical gummata in other parts of the body.

